# Vouchers in Fragile States: Reducing Barriers to Long-Acting Reversible Contraception in Yemen and Pakistan

**DOI:** 10.9745/GHSP-D-15-00308

**Published:** 2016-08-11

**Authors:** Luke Boddam-Whetham, Xaher Gul, Eman Al-Kobati, Anna C Gorter

**Affiliations:** a Options Consultancy Services, London, UK; b Marie Stopes Society Pakistan, Karachi, Pakistan; c Options Consultancy Services, Sana’a, Yemen; d Independent consultant, Roosendaal, Netherlands

## Abstract

In conflict-affected states, vouchers have reduced barriers to reproductive health services and have enabled health programs to use targeted subsidies to increase uptake of specific health services. Vouchers can also be used to channel funds to public- and private-service providers and improve service quality. The Yamaan Foundation for Health and Social Development in Yemen and the Marie Stopes Society (MSS) in Pakistan—both working with Options Consultancy Services—have developed voucher programs that subsidize voluntary access to long-acting reversible contraceptives (LARCs) and permanent methods (PMs) of family planning in their respective fragile countries. The programs focus on LARCs and PMs because these methods are particularly difficult for poor women to access due to their cost and to provider biases against offering them. Using estimates of expected voluntary uptake of LARCs and PMs for 2014 based on contraceptive prevalence rates, and comparing these with uptake of LARCs and PMs through the voucher programs, we show the substantial increase in service utilization that vouchers can enable by contributing to an expanded method choice. In the governorate of Lahj, Yemen, vouchers for family planning led to an estimated 38% increase in 2014 over the expected use of LARCs and PMs (720 vs. 521 expected). We applied the same approach in 13 districts of Punjab, Khyber Pakhtunkhwa (KPK), and Sindh provinces in Pakistan. Our calculations suggest that vouchers enabled 10 times more women than expected to choose LARCs and PMs in 2014 in those areas of Pakistan (73,639 vs. 6,455 expected). Voucher programs can promote and maintain access to family planning services where existing health systems are hampered. Vouchers are a flexible financing approach that enable expansion of contraceptive choice and the inclusion of the private sector in service delivery to the poor. They can keep financial resources flowing where the public sector is prevented from offering services, and ensure that alternative sources are available for reproductive health services such as family planning. Programs should consider using vouchers in fragile states to facilitate access to family planning services and support the countries’ health systems.

## Abstract (precis)

Vouchers for family planning in Pakistan and Yemen reduced barriers, such as cost and availability, and encouraged public and private providers to improve skills, leading to an increase in uptake of long-acting reversible contraceptives and permanent methods.

## Teaser

Vouchers for family planning in Yemen and Pakistan reduced barriers, such as cost and availability, and encouraged public and private providers to improve skills, leading to an increase in uptake of long-acting reversible contraceptives and permanent methods.

## INTRODUCTION

Many of the poorest countries are also beset by conflict or social and political unrest that challenge programs providing health services to their populations. This appears to be the case particularly in 2015 and 2016, with many conflicts across the Middle East and chronic unrest in countries such as the Democratic Republic of the Congo and Pakistan. Official development assistance is increasingly focused on these fragile states because their humanitarian needs are great and their insecurity can spread across a region and affect countries across the globe.^1^

The challenges to delivering health services in these contexts are numerous. Even where there is not full-scale conflict, governments struggle to provide health services including family planning services. Large populations are often beyond easy reach of a public health facility, and even where those facilities exist, they are under-resourced and their staff often lack the skills to provide the most effective long-acting reversible contraceptives (LARCs) and permanent methods (PMs) of family planning.^2^ Conflict exacerbates these challenges. Government systems break down, financial flows are curtailed, and basic supplies become scarce.

In the context of state fragility and conflict, service provision by the public sector is often weak; private-sector service providers can sometimes fill the gap. In Afghanistan, for example, the private sector provides 80% of all health care.^3^ In sub-Saharan Africa, as much as 60% of health expenditure is in the private sector, and in South Asia as much as 80% is accounted for by out-of-pocket costs at private-sector facilities.^3^^,^^4^ Furthermore, in these contexts, international agencies often bypass the public sector to fund urgent health services through nonprofit agencies for quick and visible results.^5^ Cost can therefore be a major barrier in seeking health services for people living in low-income, fragile environments.

To address these challenges, several organizations, such as the United Nations Population Fund (UNFPA) in Syria, are using vouchers to address the need for reproductive health services in fragile environments.^6^ Vouchers are not a new approach in conflict-affected regions—they are widely used for food, clothing, and shelter—but their use for the provision of health services in fragile contexts is a recent development (although health vouchers have been used in development aid since the 1960s).^7^

This article explores the innovative use of a demand-side financing approach in the form of vouchers to reduce barriers and catalyze uptake of family planning in Yemen and Pakistan, ranked eighth and tenth, respectively, in the Fragile States Index in 2014. ^8^ In these challenging environments, women encounter multiple barriers to choosing voluntary LARCs and PMs, evidenced by low utilization rates. Barriers to accessing LARCs and PMs are greater than those for short-acting methods. Providers may lack or have limited knowledge and skills, lack supplies, or be biased against LARCs and PMs due to the greater effort and time needed to provide them. Client barriers include costs, distance to the nearest provider, lack of information, fear of side effects, and cultural beliefs. In fragile states, supplies of implants and intrauterine devices (IUDs) are often erratic or unavailable in rural facilities due to their low priority and security problems. Clients therefore need to travel farther and incur more costs. This is compounded by insecurity, which makes longer travel unsafe. Vouchers reduce these barriers, facilitating access to the full range of family planning methods necessary for women to make an informed choice on the method to be used.

This article explores the innovative use of a demand-side financing approach to reduce barriers and catalyze uptake of family planning in Yemen and Pakistan.

We first describe Yemen and Pakistan and their voucher programs. We then use data from 2014 to show that vouchers have reduced barriers and increased uptake of family planning services. Finally, we argue that vouchers are well suited to the uncertainties of working in fragile contexts because they enable access to services where the public sector faces challenges from other priorities. Vouchers are flexible enough to keep services available despite the challenges of conflict, and they provide the most needy populations with access to services.

## COUNTRY CONTEXT

### Yemen

Yemen’s health system is dominated by the public sector in rural areas and has a strong private sector in urban areas. Family planning services in the public sector are provided for free; however, due to problems in the supply chain, they are often unavailable in lower-level facilities. In hospitals, clients are required to pay or provide missing commodities and supplies themselves.

The 2013 National Health and Demographic Survey found that knowledge of family planning is almost universal.^9^ However, the national contraceptive prevalence rate (CPR) for modern methods stands at only 29.2% (urban 40.2%, rural 24.0%) and for LARCs/PMs at 8.9% (urban 15.1%, rural 5.9%) (Table 1). The CPR for LARCs/PMs in Lahj governorate (where the voucher program operates) is less than half the national rate. In Yemen, LARCs and PMs are predominantly used in larger cities and by the wealthiest quintile (15.9%), compared with a rate of only 1.6% among the poorest quintile. Just over half (53.0%) of women obtain their contraceptive methods from the public sector, compared with 44.3% from the private sector and 1.2% from NGOs. The final 1.5% represents missing data.

**TABLE 1.** Contraceptive Prevalence Rates (%) in Yemen Nationally and in Lahj Governorate, 2013

	Yemen	Lahj Governorate
Short-acting methods		
Pills	11.6	19.7
Injectables	4.2	2.3
LAM	4.0	4.1
Condoms	0.5	0.3
*Subtotal*	*20.3*	*26.4*
LARCs and PMs		
Implants	0.6	0.5
IUD	5.9	1.8
Female sterilization	2.3	1.5
Male sterilization	0.1	0.2
*Subtotal*	*8.9*	*4.0*
**All modern methods**	**29.2**	**30.4**

Abbreviations: IUD, intrauterine device; LAM, Lactational Amenorrhea Method; LARCs, long-acting reversible contraceptives; PMs, permanent methods.

Source: Yemen National Health and Demographic Survey 2013.^9^

Discontinuation rates are high (43.0% stop using methods within 12 months), with 27.2% discontinuing for a method-related reason (e.g., method failure, side effects, inconvenient use, costs). More than a quarter (28.7%) of married women of reproductive age (MWRA) have an unmet need for family planning (15.0% for spacing and 13.8% for limiting). Unmet need for family planning in the poorest quintile is 43.1%, much higher than for the wealthiest quintile, 18.0%.^9^ Only 63% of births were wanted in 2013. If all unwanted births could be prevented, the total fertility rate in Yemen would be 3.1 instead of 4.4 children per woman.

The low family planning rates, the high unmet need, and the pronounced differences between poor and wealthy and between rural and urban areas reflect serious difficulties in family planning access. LARCs and PMs are not easily available in the public sector. In 2014, implants could only be provided by doctors, most health staff in rural public health units were insufficiently trained in IUD insertion, and sterilizations were done only in larger hospitals. Provision of LARC/PM services by the private sector in Lahj is also limited and mostly concentrated in urban areas, and accessing such services through the private sector is beyond the budget of most women.^10^

Since March 2015, the security situation in Yemen has deteriorated severely and government funds have all but stopped flowing. Funding from the central government to the governorates has been erratic.

### Pakistan

Pakistan presents a similar picture to Yemen. The public health sector provides the bulk of family planning services in rural areas, and the private sector dominates in urban areas.

The national CPR for modern methods is 26.2%. Voluntary female sterilization is the most common method at 8.7%, and thus accounts for about one-third of all contraceptive use nationally.^11^ See Table 2 for national, urban, and rural prevalence rates.

**TABLE 2.** Contraceptive Prevalence Rates (%) in Pakistan, by Urban and Rural Area and Province, 2012–2013

	Pakistan (nationally)	Urban Pakistan	Rural Pakistan	Punjab Province	KPK Province	Sindh Province
Short-acting methods						
Condoms	8.8	14.8	5.8	9.9	7.0	8.0
Pills	1.6	1.5	1.6	1.1	2.7	1.8
Injectables	2.8	2.5	2.9	2.0	5.2	3.3
LAM	1.5	0.6	2.0	2.3	0.6	0.2
*Subtotal*	*14.7*	*19.4*	*12.3*	*15.3*	*15.5*	*13.3*
LARCs and PMs						
Implants	0.2	0.1	0.2	0.2	0.0	0.3
IUD	2.3	2.6	2.2	2.9	1.5	1.2
Female sterilization	8.7	9.6	8.2	10.2	2.4	9.7
Male sterilization	0.3	0.4	0.2	0.4	0.0	0.1
*Subtotal*	*11.5*	*12.7*	*10.8*	*13.7*	*3.9*	*11.3*
**All modern methods**	**26.2**	**32.1**	**23.1**	**29.0**	**19.4**	**24.6**

Abbreviations: IUD, intrauterine device; KPK, Khyber Pakhtunkhwa; LAM, Lactational Amenorrhea Method; LARCs, long-acting reversible contraceptives; PMs, permanent methods.

Source: Pakistan Demographic and Health Survey 2012-13.^11^

The overall prevalence of LARC use in both rural and urban areas is extremely low at around 2.5%. While the overall CPR in Pakistan has increased by 9 percentage points over the last decade, the proportion of LARC users has remained almost unchanged.^11^

Two-thirds of women choosing voluntary female sterilization receive services in the public sector, while close to half of all women who adopt the IUD receive services at private-sector facilities (Table 3). Over 20.1% of MWRA continue to have an unmet need for family planning; 8.8% for spacing and 11.3% for limiting births. Unmet need is higher in rural areas (21.6%) compared with urban areas (17.1%).^11^ Barriers to adoption of family planning and continued use include an inability to pay for services and challenges to accessing services, such as low availability of skilled service providers at both public and private facilities and women’s limited mobility.^12^

**TABLE 3.** Source of Contraceptive Services (%) in Pakistan, 2012–2013

Source	Female Sterilization	IUD	Pills^a^	Injectables	Condoms^a^	Total
Public	66.5	53.3	46.5	56.3	17.7	45.6
Private	33.5	46.7	49.6	43.4	66.6	54.4

Abbreviation: IUD, intrauterine device.

a Percentages of public and private sources for pills and condoms do not total to 100 because some sources, such as shops, friends, and traditional birth attendants, are not shown.

Source: Pakistan Demographic and Health Survey 2012-13.^11^

According to World Bank data (data.worldbank.org), Pakistan allocated just 1% of gross domestic product (GDP) to education and health in 2013. Perhaps as a result, Pakistan leads the region in the highest proportion of out-of-pocket spending on health care, accounting for nearly 85.0% of total health expenditure. Health functions are distributed among several ministries at the federal and provincial levels, resulting in fragmentation. There is an absence of effective governance mechanisms; limited coordination between stakeholders; little focus on and investment in family planning and reproductive health services; failure to develop specific advocacy and information messages around family planning; and limited monitoring, evaluation, and research support for family planning and reproductive health services at the state level. High levels of poverty and illiteracy as well as lack of access to social services in rural areas compound the challenge of addressing the low CPR.

## BACKGROUND ABOUT VOUCHERS

Voucher programs have a long history of increasing access to health services among underserved populations by reducing financial and other barriers to accessing services.^13^ They can be used for a range of services but have been most commonly applied to improving access to reproductive health services such as family planning and safe motherhood (the Yemen program covers both).

Vouchers can be used for a range of services but have been most commonly applied to improving access to reproductive health services such as family planning and safe motherhood.

Voucher programs are flexible in their design, effective at leveraging the capacity of both public and private providers of health care, and can be adapted to changing situations and contexts. These are aspects that make them an effective tool for enabling access to health services in fragile states.

Voucher programs can help to strengthen health systems by targeting subsidies to overcome financial and other barriers to accessing health services and encouraging providers to improve the quality of their services for both voucher and non-voucher clients.

Voucher programs encourage improvements in quality by assessing providers in both the public and private sectors and only contracting with those who reach minimum standards or are the best available. Those who are below the minimum standards can be given a chance to improve and be contracted at a later stage. If the vouchers accompany a social health franchise, which is the case for the Pakistan program, the franchise assists the provider to reach the required quality level. Voucher programs also channel funds (voucher reimbursements) to service providers, which can be used to improve service quality (e.g., supplies and commodities, equipment, staff, and infection prevention). Furthermore, in order to attract more voucher clients and earn more, providers raise the quality of their services and become more responsive to the needs of their clients.

Vouchers enable clients to access services through both the public and private sectors without incurring out-of-pocket expenditures at the point of service, therefore bringing greater benefit to the poor. This can be amplified by making the poor or underserved priority populations for vouchers.

A voucher program is a tool that can reduce barriers for both provider and client. Vouchers level the playing field for voluntary use of LARCs and PMs. The additional income that vouchers provide, by increasing demand, motivates health care providers to offer those services and enables program managers to obtain training for their staff, where relevant, and buy supplies, if necessary. When vouchers are distributed in the community, clients receive face-to-face information and health education on LARCs and PMs and where they can be obtained. Furthermore, the voucher reduces costs for the client and can also reduce distance by encouraging an increased number of health care providers to offer LARC and PM services.

## PROGRAM DESCRIPTION

Marie Stopes Society (MSS) in Pakistan and the Yamaan Foundation for Health and Social Development in Yemen—both working with Options Consultancy Services—have developed voucher programs to increase access to LARCs and PMs.

**FIGURE 1.**
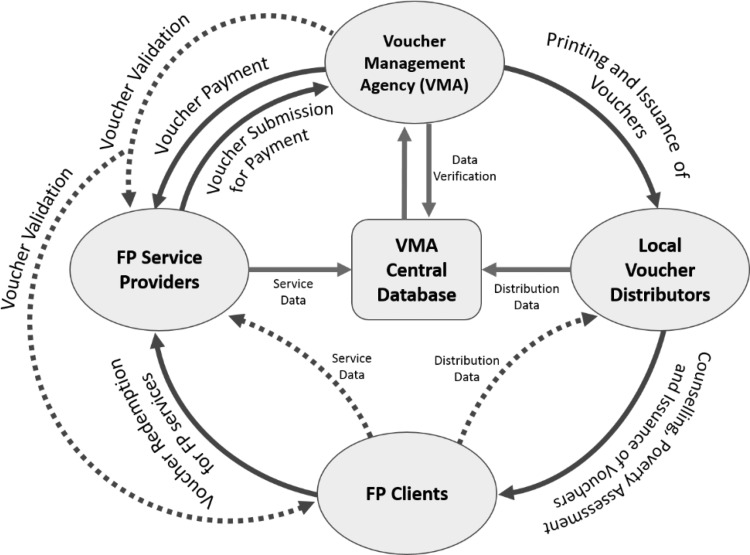
Voucher Movement and Funds Flow Abbreviations: FP, family planning; VMA, voucher management agency.

Figure 1 shows a diagram of a voucher program. A voucher management agency (VMA) receives funding and contracts with family planning service providers. The VMA prints the vouchers and issues them to local voucher distributors who visit rural communities and go door to door to counsel potential family planning clients and hand out the vouchers. The client redeems the voucher through the service provider. The provider sends a claim to the VMA for payment for a service. The VMA checks and vets the claim for completeness and consistency of the relevant data filled by the provider, and then sends payment to the provider. Payments take between 6 weeks (MSS) and 10 weeks (Yamaan) to be processed.

Table 4 describes similarities and differences between the 2 country programs. In both countries, the VMA is an NGO. The VMA recruits service providers and trains them in administration of the voucher program. Where gaps are identified, the VMA facilitates technical training of staff on service provision, such as insertion and removal of IUDs and implants. In both countries, Marie Stopes offers training on LARCs. The VMA enters into agreements with service providers; the agreements set out the detailed conditions for participation in the voucher scheme, including maintaining quality standards and setting reimbursement prices for services provided. In both countries, the VMA organizes the quality assurance. In Pakistan, supplies are provided through the voucher program; in Yemen, providers can buy supplies from a social marketing program.

**TABLE 4.** Key Aspects of the Voucher Programs in Yemen and Pakistan

Aspects	Yemen	Pakistan
Geographical area	Urban and rural areas in 2 governorates	Rural areas in 13 districts in 3 provinces
Type of VMA	NGO	NGO
Type of family planning service providers	Public and private	Private (franchised network)
Voucher distribution	Local voucher distributors paid according to number of vouchers distributed and redeemed	Local voucher distributors who receive a monthly stipend
Identifying the priority poor population	Geographical targeting of poor areas	Means testing of socioeconomic status
Cost of voucher	Free (for family planning)	Free (for family planning)
Voucher package	Counseling and LARCs/PMs (including follow-up and removal)	Counseling and LARCs/PMs (including follow-up and removal)

Abbreviations: LARCs, long-acting reversible contraceptives; PMs, permanent methods; VMA, voucher management agency.

In both countries, local voucher distributors identify eligible women in the communities, provide them with information on family planning (relevance and range of methods available, including LARCs and PMs), and hand out the vouchers for free. The voucher gives access to free family planning counseling at a health care facility as well as free LARC/PM services, while short-acting methods are provided for free or at a heavily subsidized rate.

In Yemen and Pakistan, local voucher distributors identify eligible women, provide them with information on family planning, and hand out the vouchers for free.

In both countries, program staff carry out periodic field visits to clients, during which they observe where and to whom vouchers are being distributed.

### Yemen Voucher Program

The Yemen reproductive health voucher program operates in 2 governorates, Lahj in the south and Ibb in the center. We decided to implement a voucher program based on a detailed feasibility study funded by the KfW Development Bank, which now funds the program. In this article, we describe the results for Lahj, which we selected because it is one of the poorest governorates in the south, as evidenced by the current critical food insecurity level.^14^ The governorate, however, has a fairly functional public health system that is interested in developing new approaches to reach its underserved population.

The Yemen Reproductive Health Voucher Programme runs predominantly through the public sector and in close coordination with the governorate and national ministries of health. Providers range from public hospitals to rural health units and community midwives. A substantial minority of the facilities enrolled, however, are private-sector providers.

The free family planning vouchers entitle women to a free family planning method of their choice, follow-up for any complications, and a removal if and when required. A second type of voucher is sold at a subsidized price and covers safe motherhood services. The Ministry of Health requested that the program distribute the family planning voucher for free, as it is considered a priority, underutilized service.

### Pakistan Voucher Program

The Pakistan voucher program currently covers 29 districts across 3 provinces of the country. The program started in December 2012 with 2 districts. Scaling up occurred in phases: in 2014 the program covered 13 districts in 3 provinces.

**Figure:**
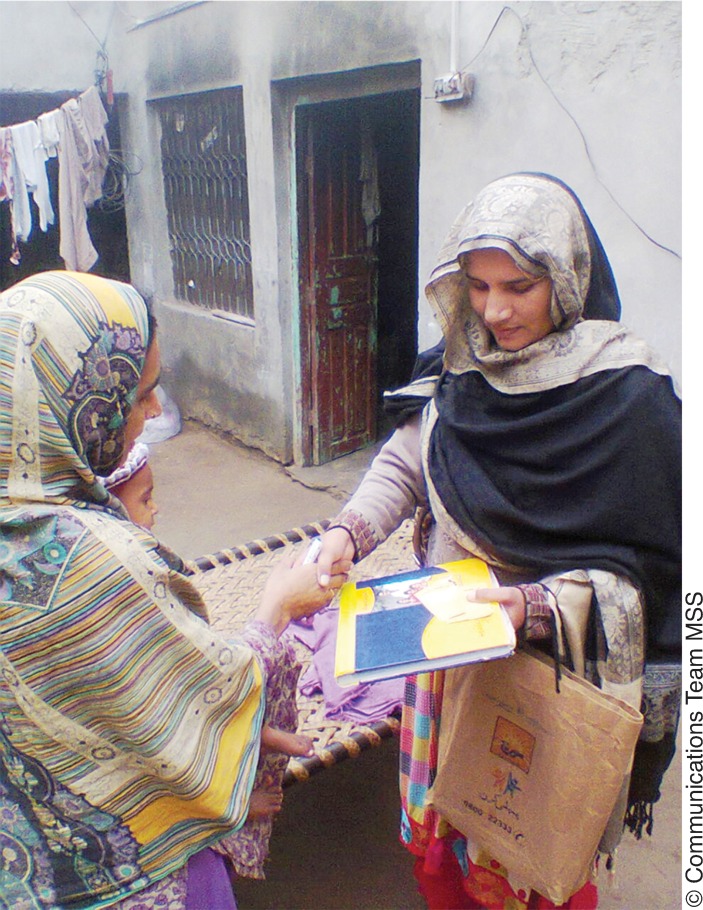
A distribution agent gives a family planning voucher to a woman in rural Pakistan.

The Pakistan voucher program functions against a backdrop of political instability, security concerns, and little focus of the government on human rights and poverty alleviation. In this challenging context, vouchers appear to strengthen the overall health system by engaging and improving the service quality of private-sector providers and by increasing the overall demand for family planning services. The Pakistan program is funded by the UK Department for International Development (DFID). We decided to implement a voucher program based on vouchers’ known efficacy in decreasing access barriers and increasing uptake of targeted services, and their adaptability to challenging operating environments.

The Pakistan Reproductive Health Franchise (RHF) program works entirely with private providers in a social franchise network, called the Suraj Network. While some of these providers are medical doctors, the majority are nurses and lady health visitors, who are a paramedic cadre with a 2-year diploma, specifically trained to provide family planning and reproductive health services.

**FIGURE 2.**
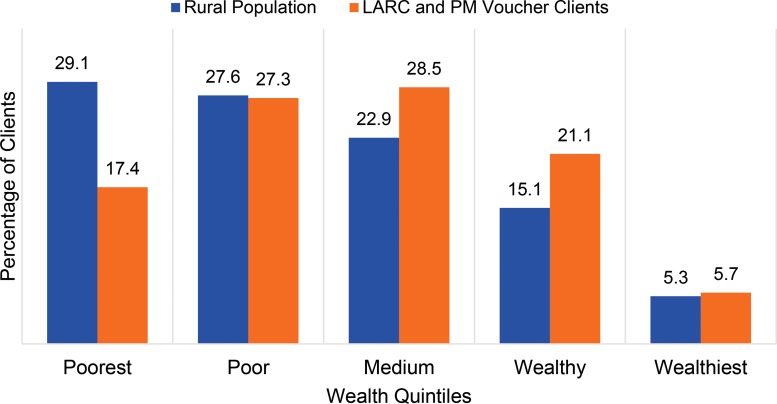
Wealth Index Distribution of the Rural Population in Pakistan and of MSS Voucher Clients for LARCs and PMs (N=1,557), 2014 Abbreviations: LARCs, long‐acting reversible contraceptives; MSS, Marie Stopes Society; PMs, permanent methods. Source: Wealth index distribution of the rural population from the 2012-13 Pakistan Demographic and Health Survey^11^; of voucher clients, from the MSS annual client exit interview survey conducted in December 2014.

From an equity perspective, vouchers are considered to be effective in increasing access to family planning services, especially for the poor. Figure 2 presents information from the Annual Client Exit Interview survey conducted in 2014 with clients receiving family planning services at MSS social franchise facilities. Overall, a total of 1,557 family planning clients were interviewed at 151 MSS social franchise facilities in December 2014. The wealth index analysis is based on the weighted analysis. The wealth distribution of clients who access family planning services using a free voucher was similar to the wealth index of rural Pakistan,^11^ with a slightly higher number of voucher clients in the wealthier quintiles.

A higher proportion of Suraj voucher clients fell within the second and third wealth quintiles, rather than the first and second quintiles. This is not because the poor are not effectively identified as an underserved population; rather it is explained by the fact that, in addition to eligibility being determined by a poverty-ranking tool, vouchers are distributed to clients as a function of their capacity-to-benefit (i.e., a woman is given a free service voucher if she reports lack of access to finances, irrespective of what her poverty-ranking score is).

## METHODS AND RESULTS

Voucher programs, because they pay based on results, have strong built-in data collection and analysis mechanisms. Data on targeted services are collected, and trends in service utilization, are analyzed as an integral part of voucher monitoring systems. These data can be compared with historical data to evaluate the effect vouchers have on utilization.

### Yemen

In Lahj governorate, the program was scaled up in phases from April 2013 to September 2014 when all 15 districts were finally participating. From April 2013 to April 2015, the voucher distributors handed out around 56,000 family planning vouchers. There was no requirement to use the voucher immediately; distributors explained that recipients might use them at a later date. Data up to September 2015 show that recipients redeemed about 12,000 vouchers (1,135 for LARCs/PMs and the rest for counseling and/or short-acting methods). The war caused serious problems in the supply chain, and many providers closed their facilities temporarily, which resulted in a 21.0% redemption rate rather than the expected rate of 40.0%.

For Lahj, we estimated the expected annual uptake of LARCs/PMs among eligible MWRA needed to maintain the CPR. We used population data for 2004,^15^ corrected for population growth, as well as the CPR from the recent 2013 National Health and Demographic Survey.^9^ The formula we used is as follows: MWRA multiplied by the annual CPR for each method.

Long-acting methods provide multiple couple-years of protection (CYP).^16^ A crude but indicative formula to obtain the annual CPR for a method is to divide its CPR by the CYP. Table 5 presents the annual CPR for implants, IUDs, and female sterilization as well as the number of women that would need to be added annually to maintain the current CPR. The expected annual number of women choosing LARCs or PMs in Lahj is 840. However, because the program did not reach full scale until September 2014, this number had to be corrected for the period that eligible MWRA had access to vouchers that they could use. We calculated this number by using only the person-months that eligible women were covered by vouchers in 2014. This was 62.0% of all person-months in 2014. Therefore, the expected number of women choosing LARCs or PMs, among eligible women covered by a voucher, is 62.0% of 840  =  521. We compared this result against a year of service data generated by the vouchers (Table 6). We found a much higher-than-expected uptake of LARC and PM services in voucher areas in the year 2014: 720 vs. 521 expected overall. This 38.0% difference is a strong indication of the positive effect of the family planning vouchers.

We found a much higher-than-expected uptake of LARC and PM services in voucher areas in 2014: 720 vs. 521 expected.

**TABLE 5.** Estimated Annual LARC and PM CPR and Expected 2014 Annual LARC and PM Uptake Among MWRA, Lahj Governorate, Yemen

	CPR (%)	CYP per Unit	Annual CPR^a^ (%)	Expected 2014 Annual Uptake of LARCs/PMs^b^ (No. of Women)
Implants	0.5	3.2	0.156	188
IUD	1.8	4.6	0.391	471
Female sterilization	1.5	10.0	0.150	181
**LARCs or PMs**	**3.8**		**0.697**	**840**

Abbreviations: CPR, contraceptive prevalence rate; CYP, couple-years of protection; IUD, intrauterine device; LARC, long-acting reversible contraceptive; MWRA, married women of reproductive age; PM, permanent method.

a To obtain the annual CPR, we divided the CPR from the 2013 National Health and Demographic Survey^9^ by the CYP.

b To obtain the expected annual uptake of LARCs/PMs, we multiplied the population of MWRA (120,478) in 2004 by the annual CPR for each method.

**TABLE 6.** Number of LARC and PM Services Provided Through the Yemen Voucher Program in Lahj Governorate, 2014

	Expected No. of Women Choosing LARCs or PMs in 2014	No. of Women Choosing LARCs or PMs Through Vouchers in 2014	Percentage Difference
Implants	117	273	133.3
IUD	292	428	46.6
Female sterilization	112	19	-83.0
**Total**	**521**	**720**	**38.2**

Abbreviations: IUD, intrauterine device; LARC, long-acting reversible contraceptive; PM, permanent method.

It should be noted that this is *at least* a 38.0% difference. Not all LARC and PM service provision was through vouchers, because non-voucher clients also accessed LARCs and PMs. Service providers reported that the number of non-voucher clients was approximately the same as the number of voucher clients. Not all MWRA took vouchers. By the end of December 2014, distributors had handed out approximately 44,000 family planning vouchers, representing around one-third of all MWRA in Lahj. Furthermore, not all private-service providers were contracted under the voucher program. In 4 of the 15 districts, not all women were eligible; we used an approach that gave priority to poor households in these districts (as opposed to setting priorities geographically in the remaining districts), which excluded a large group of non-poor women. Finally, of the expected 409 implant and IUD removals in 2014, the program provided only 96 removals. This suggests that many women went elsewhere to remove or replace their methods, and that many of those using a voucher to access LARCs and PMs were new users.

The use of implants was more than double the use we would have expected among eligible women in the absence of vouchers; for the IUD, this was 46.6% more. The lower number of sterilizations merits further study. One of the reasons might be that, in practice, with implants and the IUD becoming more available, women preferred these methods over sterilization. Also, as noted above, not all LARC and PM service provision was through vouchers and not all service providers were contracted under the voucher program.

Although the CPR for modern methods has increased considerably during the last decade— from 13.0% in 2003, to 19.0% in 2006, to 29.2% in 2013 nationally and to 30.4% in Lahj^9^—most women use short-acting methods. There have been some small-scale but notable efforts to increase uptake of LARCs and PMs, such as Marie Stopes International Yemen’s social franchising of private midwives (the Rayaheen program), but the voucher program is the only substantial intervention implemented at scale in Lahj.

### Pakistan

In 2014, the Pakistan program covered 13 districts with 113 franchised private providers. The priority population around each franchised clinic is estimated at 30,000; the total priority population in these 13 districts was 3,390,000, with 474,600 MWRA. We calculated the expected number of MWRA accessing LARCs or PMs using the same formula applied to the Yemen data (Table 7). Table 8 compares the expected number of women accessing LARCs or PMs against a year of service data generated by the voucher program in Pakistan.

**TABLE 7.** Estimated Annual LARC and PM CPR and Expected 2014 Annual LARC and PM Uptake Among MWRA, in 13 Pakistan Program Districts

	CPR (%)	CYP per Unit	Annual CPR^a^ (%)	Expected 2014 Annual Uptake of LARCs/PMs^b^ (No. of Women)
Implants	0.2	3.2	0.06	285
IUD	2.2	4.6	0.48	2,278
Female sterilization	8.2	10.0	0.82	3,892
**LARCs or PMs**	**10.6**		**1.36**	**6,455**

Abbreviations: CPR, contraceptive prevalence rate; CYP, couple-years of protection; IUD, intrauterine device; LARC, long-acting reversible contraceptive; MWRA, married women of reproductive age; PM, permanent method.

a To obtain the annual CPR, we divided the CPR from the 2012-13 Demographic and Health Survey^11^ by the CYP.

b To obtain the expected annual uptake of LARCs/PMs, we multiplied the population of MWRA (474,600) in 2004 by the annual CPR for each method.

**TABLE 8.** Number of LARC/PM Services Provided Through the Pakistan Voucher Program, 2014

	Expected in 2014	Total 2014 Through Vouchers	Percentage Difference
Implants	285	3,826	1,242%
IUD	2,278	67,750	2,874%
Female sterilization	3,892	2,063	-47%
**LARCs/PMs**	**6,455**	**73,639**	**1,041%**

Abbreviations: IUD, intrauterine device; LARC, long-acting reversible contraceptive; PM, permanent method.

We estimate that the number of women accessing LARCs or PMs in the priority areas in 2014 was approximately 10 times more than expected without vouchers (73,639 vs. 6,455, respectively). While demand for family planning has been increasing over the last decade, provision of family planning services through the government has lagged behind and is low (Table 3). In the priority population described above, 102,514 MWRA (21.6%) had an unmet need for family planning.^11^

**FIGURE 3.**
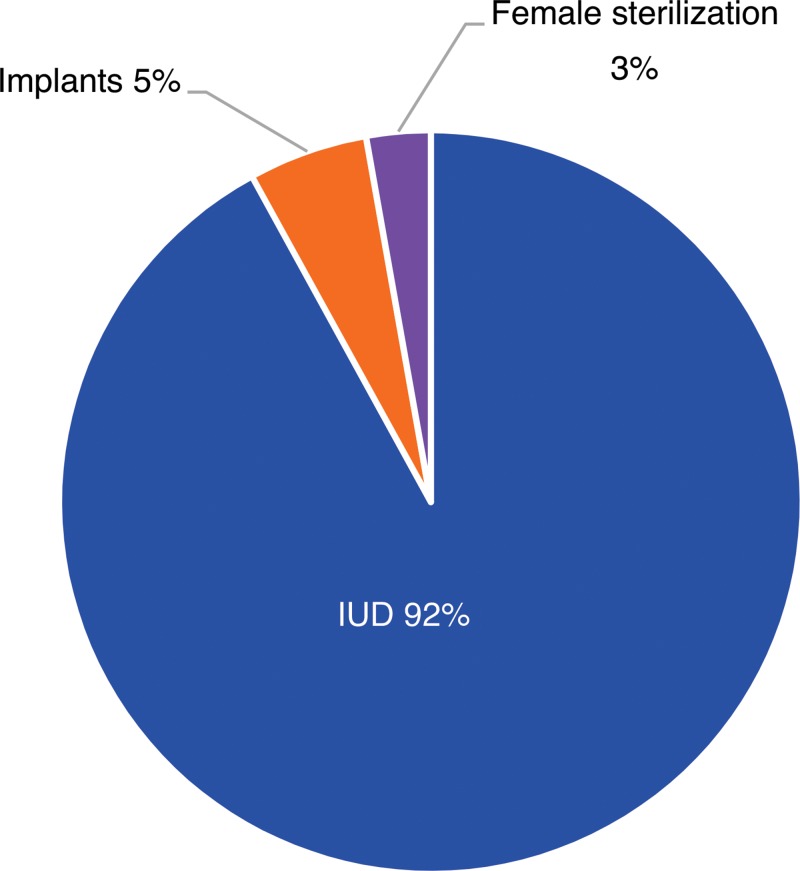
Vouchers Redeemed in Pakistan by Contraceptive Method, 2014

Over 88% of clients who opted for LARCs or PMs used a voucher. Field workers distributed a total of 83,920 vouchers across the 13 districts in the 3 provinces with an overall redemption rate of 87.8%. The redemption rate was the highest in Punjab at 96.0%, followed by Sindh and Khyber Pakhtunkhwa (KPK) at 90.0% and 65.0%, respectively (Table 9). IUDs were by far the preferred choice of clients, accounting for 92% of the vouchers used (Figure 3).

**TABLE 9.** Voucher Distribution and Redemption by Province in Pakistan, 2014

Province	Vouchers Distributed No. (%)	Vouchers Redeemed No. (%)	Redemption Rate (%)
Punjab	52,249 (62)	49,969 (68)	95.6
KPK	18,989 (23)	12,304 (17)	64.8
Sindh	12,682 (15)	11,366 (15)	89.6
**Total**	**83,920 (100)**	**73,639 (100)**	**87.7**

Abbreviation: KPK, Khyber Pakhtunkhwa.

The free LARC and PM services provided through the vouchers, the marketing and health education by the voucher distributors at a woman’s doorstep, and the higher quality of the franchised clinics appear to have contributed to this huge success in reaching poor women in rural areas of Pakistan.

Because of the historically poor trends in the availability and uptake of implants and IUDs, it may be safely assumed that, in the absence of demand- and supply-side efforts to improve awareness of family planning and the supply and quality of LARC and PM services, uptake would have been considerably lower. Interventions to reduce barriers to contraceptive access are needed in Pakistan’s rural areas, and vouchers are an approach to achieving that goal.

Interventions to reduce barriers to contraceptive access are needed in Pakistan’s rural areas, and vouchers are an effective approach to achieving that goal.

We were surprised by the extent of the impact of vouchers in Pakistan on family planning uptake. To verify this result, the RHF Pakistan program is conducting a multicluster study to evaluate the impact of family planning service vouchers when combined with social franchising and different behavior change approaches.

## DISCUSSION

From our experience with voucher programs in Yemen and Pakistan, we have observed that vouchers alone and vouchers combined with social franchising facilitate access to services when traditional supply-side approaches are unable to do so.

When the Yemen voucher program started, very few facilities were offering LARC or PM services. The incentives offered by the program encouraged both public and private facilities to access training in LARCs and PMs through the local health offices and NGOs, thus increasing choice and access for women.

Despite the challenges of funding through government channels, the voucher program (which also provides safe motherhood vouchers) has continued to pay providers for the services they deliver. These are extremely valuable funds, which facilities use not only for family planning and safe motherhood services but also for other health services, such as child illness and malaria.

Fighting within Yemen shut down several key government facilities, including the main governorate hospital, for several months. However, women were able to receive reproductive health services from other facilities in nearby districts, including privately run midwife clinics and hospitals. The safe motherhood voucher also entitles women to free family planning services as part of the postnatal care package that it offers.

In Pakistan’s challenging context, vouchers were able to increase family planning uptake. Vouchers appear not only to help strengthen the overall health system by engaging and improving the service quality of private-sector providers, but they also increase the overall demand for family planning services.

Supporting our own findings, a 2011 study led by MSS evaluated the impact of vouchers on women choosing the IUD in rural Pakistani communities. MSS conducted this quasi-experimental study in 1 intervention district and 1 control district in each of the provinces of Sindh and Punjab, with 4 service providers in each district. MSS carried out a baseline survey with a sample of more than 4,000 eligible women who were followed up 18 months later. During this 18-month trial combining social franchising and family planning service vouchers, the overall CPR in the intervention districts increased from 27.2% to 48.0%, while in the control districts the CPR increased from only 28.5% to 29.7%.^17^ Thus, the net increase in the intervention area was 19.6%.

### Program Implications

There are a number of lessons that we can draw from the voucher programs in 2 different, challenging, and fragile environments.

**Figure:**
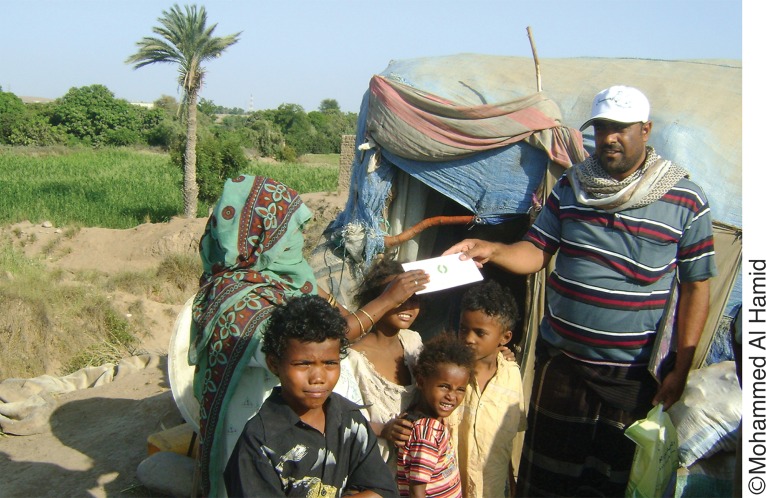
A voucher distribution agent in Yemen gives a family planning voucher to a client.

- **Voucher programs work with both the public and private** **sector** (both for-profit and nonprofit) within the health system as a whole, and can provide comprehensive family planning services by **expanding the contraceptive method choice to a larger group** and increasing the number of providers offering LARCs. By engaging the nonstate sector, voucher programs can fill gaps that under-resourced and, at times, incapacitated, government systems cannot address. The government of Pakistan provides family planning free through its broad network of rural health care providers. However, these providers do not offer LARCs or PMs because they are not fully trained in the provision of these methods and focus instead on short-acting methods, despite serious problems with ensuring a constant supply of such contraceptives. The voucher approach enables private providers who offer LARCs and PMs to be paid when providing these services to poor rural women who would otherwise not be able to afford such services. Payments incentivize these providers to offer family planning services that they otherwise would not offer because the limited numbers of clients seeking LARCs or PMs do not generate sufficient revenue for their businesses. Thus, vouchers expand the method choice. Furthermore, they act as an incentive to reach out to the poorer clients.
- **Voucher programs can keep vital funds flowing to public health care providers in times of conflict.** In Yemen in early 2015, a rebel militia from the north of the country (the Houthis) overthrew the elected government. The government was, for a time, incapacitated and funds did not flow to public health facilities. However, the Yemen voucher program continued to pay for services, providing vital income for facilities and ensuring that services continued to be available to women who needed them.
- **Voucher programs give women alternatives in places where government facilities are temporarily closed due to conflict.** As outlined previously, the voucher program has enabled women to continue to access health services in Yemen despite public facilities being closed by the ongoing conflict. By providing a mechanism through which to pay private facilities for their services, voucher programs help those facilities to fill a gap in service provision and enable women to access family planning and other key reproductive health services.
- **Voucher programs can standardize quality assurance for targeted services and incentivize providers to maintain and improve the quality of the services they provide.** In fragile contexts, ministries of health can struggle to maintain oversight for all services. Implicit in the voucher approach is regular quality assurance to ensure that participating service providers maintain minimum standards. Marie Stopes International applies its global standards for high-quality family planning to its franchise network in Pakistan. Yamaan works with the government of Yemen Quality Improvement Programme (which is supported by the Deutsche Gesellschaft für Internationale Zusammenarbeit [GIZ]), focused on improving the standard of reproductive health care. Voucher service providers use their service income to improve the quality of their services and thereby attract more clients.
- **Voucher programs are flexible and can adapt to the particular needs of the population and context in which they operate.** While voucher programs strictly adhere to certain processes and procedures (e.g., regular verification and monitoring for fraud control), their design can be adjusted to realities on the ground. For example, in Yemen during the recent crisis, the price of fuel and medical supplies increased substantially. The program was able to easily adjust its pricing policy to accommodate this, ensuring providers stayed in the program and clients continued to receive services. If necessary, voucher programs can include more or other service providers or add health services or other benefits to the voucher package. Vouchers can be combined with other financing mechanisms, such as performance-based financing and conditional cash transfers. Also, once voucher programs are set up, their priority populations can be changed, or they can be scaled up to other geographical areas. Such decisions can be made rapidly to adapt to the constantly changing environment of a country at war.

Voucher programs can keep vital funds flowing to public health care providers in times of conflict.

### Limitations

The authors adopted the formulae set out above for these calculations to look at the direction of change in contraceptive use catalyzed by vouchers. However, we recognize that this methodology could be improved to increase the accuracy of the results. We welcome suggestions from readers to improve our methodology.

## CONCLUSION

Voucher programs are not a health system approach and do not solve the many challenges faced by health systems in fragile states. They do, however, have a number of strengths. In the short term, they are a proven approach to increasing use of priority services;^18^ they improve equity; they can have a positive impact on the quality of services;^18^^,^^19^ and recent experience, described in this article, has shown that they are a useful tool for helping to maintain health services when public systems are failing to do so. In the medium to long term, vouchers accustom providers to processes akin to insurance, and they can be a precursor to health insurance programs. They also accustom providers to contracting out to the private sector. Both health insurance and contracting to the private sector are health systems solutions.

Addressing the financial barriers associated with accessing health services can have a substantial effect on utilization, even in fragile states. A 38% increase in voluntary LARC and PM use in Yemen and a 10-fold increase in Pakistan demonstrate this. We would encourage program implementers in fragile settings to consider vouchers to reduce barriers and improve access to family planning services.

## Notes

**Peer Reviewed**
